# Neural Correlates of Speech Processing in Prelingually Deafened Children and Adolescents with Cochlear Implants

**DOI:** 10.1371/journal.pone.0067696

**Published:** 2013-07-04

**Authors:** Magdalene Ortmann, Arne Knief, Dirk Deuster, Stephanie Brinkheetker, Pienie Zwitserlood, Antoinette am Zehnhoff-Dinnesen, Christian Dobel

**Affiliations:** 1 Institute for Biomagnetismus and Biosignalanalysis, University of Muenster, Muenster, Germany; 2 Jean Uhrmacher Institute for Clinical ENT-Research, University of Cologne, Cologne, Germany; 3 Department of Phoniatrics and Pedaudiology, University Hospital Muenster, Muenster, Germany; 4 Department of Psychology, University of Muenster, Muenster, Germany; University of California, San Francisco, United States of America

## Abstract

Prelingually deafened children with cochlear implants stand a good chance of developing satisfactory speech performance. Nevertheless, their eventual language performance is highly variable and not fully explainable by the duration of deafness and hearing experience. In this study, two groups of cochlear implant users (CI groups) with very good basic hearing abilities but non-overlapping speech performance (very good or very bad speech performance) were matched according to hearing age and age at implantation. We assessed whether these CI groups differed with regard to their phoneme discrimination ability and auditory sensory memory capacity, as suggested by earlier studies. These functions were measured behaviorally and with the Mismatch Negativity (MMN). Phoneme discrimination ability was comparable in the CI group of good performers and matched healthy controls, which were both better than the bad performers. Source analyses revealed larger MMN activity (155–225 ms) in good than in bad performers, which was generated in the frontal cortex and positively correlated with measures of working memory. For the bad performers, this was followed by an increased activation of left temporal regions from 225 to 250 ms with a focus on the auditory cortex. These results indicate that the two CI groups developed different auditory speech processing strategies and stress the role of phonological functions of auditory sensory memory and the prefrontal cortex in positively developing speech perception and production.

## Introduction

Cochlear implants (CI) constitute the most successful neuroprotheses developed to date. Originally designed to restore speech perception in the profoundly bilateral deaf, it is today broadly administered to postlingually or prelingually deafened, unilaterally or bilaterally hearing-impaired and completely or profoundly deaf persons. One group seems to profit most from cochlear implants: young children who were born deaf or deafened before speech development started. By receiving a CI very early in life, these prelingually deafened children later have a chance of developing normal speech performance and of freely communicating with other normal-hearing children and adults [1]. This is mostly due to the administration of well-developed speech therapies and highly sophisticated CI hardware. Nevertheless, a surprisingly strong variation in language development can be observed in prelingually deafened children with CIs. Although parts of this variation can be attributed to hearing age (number of years with auditory experience after CI surgery) and age at implantation (duration of deafness), much of this variance remains unexplained [2]–[4].

In the current study, we compare prelingually deafened children and adolescents with CIs who had developed either high or low language abilities (good vs. bad performers), even though basic hearing abilities are highly developed in both groups. We target their ability to discriminate simple speech sounds (e.g., “/bu/” vs. “/ba/”) and assess how they differ in terms of neurophysiology (evoked electro-encephalographic responses) and behavior. The main assumption behind the present study is that the auditory deprivation of sound or speech is accompanied by a deficit in representing speech stimuli with high quality over a sufficient amount of time to enable speech perception and production. This early memory trace (i.e., the ability to represent and maintain auditory stimuli) is also termed auditory sensory memory [5]. Phoneme discrimination, when explicitly measured, is taken as an index for phonological awareness that refers to the ability to detect and manipulate sounds at the level of syllables and phonemes [6]. Thus, deprivation of speech leads not only to reduced auditory sensory memory and phonological awareness but, as a consequence, also to impaired language abilities that span all levels of linguistic processing [7]–[9]. In the following, we address each of these aspects in turn.

### Phonological Awareness, Auditory Sensory Memory and Language Development in CI Users and Healthy Controls

Human languages consist of words as units of analysis that allow us to comprehend or convey an intended meaning. Words often consist of smaller units of meaning called morphemes, which in turn are composed of sequences of speech sounds – so-called phonemes (e.g., /t/ in ‘cat’, ‘stand’ or ‘water’) [10]. Although the manifestations of a specific phoneme may sound different in each word (as /t/ in the example above), they belong to the same phonemic category. A correct differentiation of phonemes is crucial in order to distinguish between words of similar sound (e.g., *bet* and *bat*). Each language uses a specific repertoire of phonemes that constitutes a subset of all of the phonemes in the world. Amazingly, human infants can categorically distinguish between all phonemes – even those that are not part of the language environment they are born into [11]–[13]. However, from six months of age onwards, their phoneme perception is altered by their exposure to their mother tongue, showing that native-language phoneme processing is supported while the ability to distinguish non-native phonemes declines [14], [15]. Phoneme discrimination abilities at this age are a reliable predictor of speech performance at the age of two years [16]. Similarly, problems in phoneme categorization and phonological awareness have been intensively discussed as causes for language dysfunctions such as Specific Language Impairment (SLI) [17] and other developmental linguistic impairments [18], [19].

Phoneme discrimination, obviously a core function in language processing, is closely connected to auditory sensory memory. As stated above, infants learn to prioritize the phonemes of their mother tongue. To do so, these must be selected from the incoming auditory signal. Within this encoding process, a memory trace is built. Unfortunately, this early memory trace, embodied in the auditory sensory working memory [20], is very unstable and prone to decay. To ensure its maintenance, phonemes are rehearsed and manipulated in phonological working memory for final storage in long-term memory [21]. Each time an infant has access to new speech signals, the presented phonemes are compared to the already stored prototypes. With each recognition, the cortical representation of the prototype is strengthened and future recognition facilitated [22]. This process leads to the superiority of native versus non-native speech sounds, which are presented less often. Additionally, when infants retrieve stored phonemes for articulation from memory, this simultaneously trains not only phoneme discrimination but also the retrieval and storage of the learned speech sounds [23].

Unfortunately, children with cochlear implants skip a substantial phase of hearing experience and, as a consequence, a possibility to train their phoneme discrimination abilities and their auditory sensory memory. It is therefore unsurprising that prelingually deafened CI users display both weaker phonological awareness and impaired auditory sensory memory (when tested, for example, via working memory performance, as in repeating lists of words) relative to normal controls [24]. This notably does not apply to visual-spatial working memory functions [25], [26]. Importantly, deficits in phoneme discrimination do not only affect the auditory working memory, but also higher-order language abilities: phoneme discrimination has a strong influence on word decoding, reading, reading comprehension [27]–[30] and communication mode [31]. It is thus likely that prelingually deafened children with CIs who substantially differ in speech performance will also show differences in phoneme discrimination and auditory sensory memory.

### Testing Phoneme Discrimination and Auditory Sensory Memory by ERPs in CI Users and Healthy Controls

In the present study, we employ event-related brain potentials (ERP) to learn about the processes underlying phoneme discrimination in good and bad performers. ERPs offer an objective and time-sensitive measurement of central auditory processing and can be recorded using non-invasive methods such as electro-encephalography (EEG) [32]. While there exist several components for studying the auditory system, such as the P50 or the N100, the Mismatch Negativity (MMN) seems particularly suited to study whether and how phonological items can be differentiated from each other. The MMN is triggered 150 to 250 ms after the appearance of a *deviant* stimulus that is randomly distributed across frequently presented *standards* [33]. It is hypothesized to reflect automatic detection of differences between the standard, which is held in auditory sensory memory, and the deviant, which is present in the current sensory input [34]. The MMN is evoked if the neural response triggered by the deviant does not match the (still available) memory trace formed by preceding standards [35]. The MMN thus mirrors the functionality of auditory sensory memory [36]. Initial research demonstrated its sensitivity to rather basic differences between auditory stimuli, but more recent research has shown that the MMN can also be evoked when standards and deviants differ in only one phonetic feature (e.g., /b/ and /p/) [37], [38] or in higher levels of speech processing such as their semantic, syntactical or lexical properties [39], [40]. In conformance with fMRI data [41], there is evidence that the MMN is not, as previously believed, solely evoked in the bilateral auditory cortex [42], [43] and the temporal lobes [44], [45], but also has prefrontal sources [46]–[49], especially in speech perception [40], [50].

In the following discussion, we briefly review several characteristics that make the MMN a well-suited tool for investigating phonological processes in special populations such as CI users. As mentioned above, the MMN can be elicited without directed attention [34], which allows for studying participants with lower attention spans, for example, by letting them watch a silent movie while their EEG is recorded. This makes it particularly suitable for investigating information processing in children [36], as it develops during infancy [51] or, specific to language, for researching the effects of intensive phonological training (in adults: [52]).

In CI users, the MMN is a good indicator of developing phoneme discrimination abilities after implantation [53] and is positively correlated with speech perception abilities [54]. Moreover, it has been shown that the MMN does not differ between postlingually deafened good performers (in terms of speech perception) and controls with normal hearing, but is absent in bad performers [55]–[57]. Note that none of these studies used complex speech stimuli and, due to their equipment and methods, could not draw conclusions about the cortical generators of the MMN.

Finally, it has also been shown that the MMN is not only sensitive to deficits in auditory sensory memory itself (for review: [5]), but also indicates that increased phonological awareness is positively correlated with phonological short-term memory [24], [58]. Thus, the MMN is sensitive to the connection of phonological processing with auditory sensory memory, a crucial functional interplay, as argued above.

In order to study phonological processing and its underlying neural sources in good and bad performers, we tested pairs of prelingually deafened cochlear implant users between seven and 19 years of age who had received their implants before their fifth birthday (with the exception of one pair). The groups differed solely in their language development: nine “good performers” achieved a very high level of speech performance, while the nine “bad performers” had a very low score. Their assignment as good or bad performers was based on group-specific linguistic criteria that assessed all sub-domains of speech perception and production. Importantly, hearing abilities were high in both groups, and age at implantation and hearing age in years were matched within each pair. All participants had to perform an auditory digit span test as a behavioral index for auditory sensory or working memory as well as several tests on phoneme discrimination. Based on the latter results, individual stimulus material for triggering the MMN in each participant was chosen. To study the neural generators underlying the MMN, we performed an analysis in source space (L2-Minimum-Norm-Estimates; [59], [60]), which is widely used in language processing [40], [61], [62] and for the localization of the MMN [63]. To date, source localization has only been rarely used in CI users [64]–[66] and never in response to speech stimuli.

On a behavioral level, we hypothesized significantly better phoneme discrimination abilities and auditory working memory functions in good performers than in bad performers, with the good performers being similar to healthy controls. Additionally, we expected differences in phonological awareness and auditory sensory memory to be reflected at a cortical level by higher neural activity of the MMN. Based on the current literature, we expected differences between CI groups in at least one of the regions associated with the MMN: the temporal (with a special focus on the auditory cortex) as well as the prefrontal cortex.

## Materials and Methods

### Participants

#### CI Users

To find suitable participants that fulfilled our predefined logopedic and phoniatric criteria, we screened 64 patient files of children with CIs, stored in the archive of the Department of Phoniatrics and Pedaudiology of the Muenster University Hospital, Germany. Our goal was to find pairs of prelingually deafened patients that could be matched according to hearing experience and age at implantation, with comparable, very good overall hearing abilities, but who clearly differed in speech performance. The group with high speech performance was labeled “good performers”, the group with low performance “bad performers”. After initial file screening, we identified potential pairs of good and bad performers that were invited to our laboratory, where a speech therapist intensively examined their spontaneous speech. This examination involved all levels of speech perception and production, including articulation, syntax, morphology and semantics. Hearing age was considered throughout. All four sub-dimensions were rated on an ordinal scale from 1 to 6. Good performers scored two or lower, while bad performers were rated at 4 or higher. There was thus no overlap in speech performance between good and bad performers. For detailed information about tests used to assess each sub-dimension and their characteristics, see Table S1. A global measure of speech performance in each CI user was defined by adding up all sub-dimensions (4–24 points). CI-users who did not fulfill the criteria for group inclusion were replaced by others. Finally, 18 subjects (9 pairs containing 8 girls and 10 boys aged 7–19 with an average of 12.9 years) were selected for further investigation. Good performers reached on average 6 points in global speech performance (range: 4–8 points; std = 1.73), bad performers 18.2 (range: 16–20; std = 1.8).

Note that, according to standard clinical testing, all children had very good hearing abilities. This was ensured by accepting only those subjects for the study who had at least 70% correct answers in the *Freiburg Monosyllable Word Test* (FMWT [67], good performers mean = 95% ±5, bad performers mean = 81% ±8; t_(8)_ = 4.5, p<.01), which assesses correct repetition of frequent monosyllabic words and thus tests basic comprehension abilities for high-frequency words without involvement of compensational processes due to a larger semantic context. Based on clinical experience, a value of 70% allows patients to have telephone calls – a milestone in the rehabilitation of CI patients. It was indeed the case that every participant in our study was able to do this. Note that accurate repetition of monosyllabic words in the FMWT is far easier than producing accurate spontaneous speech. Therefore, although bad performers had trouble with articulation in *spontaneous* speech, all participants achieved good results in the FMWT. Moreover, the MED-EL Teen-Ears test battery, established to assess hearing in children and young adults following CI implantation [68], demonstrated very good hearing abilities for all subjects. Here, CI users had to: 1) identify the number of syllables in spoken words (1, 2 or 3 syllables); 2) recognize spoken sentences from a set of their written counterparts (“sentences in closed set”); and 3) recognize key words within spoken sentences without the help of lip reading or their written counterpart (“sentences in open set”). In tests 1 and 2, both groups had 100% correct answers; in test 3, good performers achieved 99%±1.6 and bad performers 90%±10.8 correct answers.

To enable the best possible quality of auditory transmission, the speech processors of the CI users were fitted for optimal transmission of the incoming signal to the auditory system, especially in the dynamic range of speech signals between 35 dB and 65 dB. This was ensured by a measurement of the aided thresholds. The hearing threshold should be at around 25–30 dB to inhibit low level background noise and no uncomfortable loud impressions of sounds should emerge to avoid harmful signal peaks in everyday life. The aided thresholds were measured in the frequency range of the CI system between 250 Hz and 8000 Hz with third octave noise. The presentation of the sounds started at 60 dB and decreased in steps of 20 dB until the patient did not hear any sounds. The loudness then increased by 5-dB steps until the patient reported hearing again. This threshold value was controlled by variation of 5 dB steps below the first measured threshold. After determining the hearing thresholds of all frequencies, the volume was increased until the patient reported uncomfortable loudness or until 100 dB was reached. Thus, it was ensured in each patient that the physiological dynamic range was maximally used without leading to distortions.

In short, using this intensive screening procedure, we established that the two groups had no overlap in their performance for speech perception and production (tested via global speech performance: t_(8)_ = 14.4, p<0.001, N = 18). Importantly, both groups had equal hearing experience, were exposed to the same duration of deafness in years and had high hearing abilities.

All subjects deafened prelingually and were provided with hearing aids. All except one pair received their CI before their fifth birthday. The two members of this pair were offered CIs, too, but parents decided to continue with high-level hearing aids first before later switching to cochlear implants.

As mentioned above, CI users were initially matched according to hearing age and age at implantation by rounding to whole years. This resulted in no difference between groups in age at implantation (t_(8)_ = −0.55, p = 0.59) or hearing age (t_(8)_ = 1.35, p = 0.21). The same was true when the exact age was taken into account (years; months): hearing age (t_(8)_ = 1.12, p = 0.27); age at implantation (t_(8)_ = 0.59, p = 0.57). However, if the outlier pair 9 was excluded from analyses, age at implantation was significantly lower in good performers (mean = 2.5 years, std = 0.81) than in bad performers (mean = 3.4 years, std = 1.11; t_(7)_ = 3.87, p = 0.006). To test the relationship between age at implantation and global speech performance, these variables were correlated. Including pair 9, age at implantation again had no influence on global speech performance (r = 0.125, p = 0.622). By excluding pair 9, the correlation test showed a trend towards significance (r = 0.49, p = 0.053), indicating that early CI implantation correlated with higher speech development. To assess its potential influence on experimental results, we included age at implantation as an additional variable in all relevant steps of data analyses. For further information about participant data and descriptive statistics, see Table 1.

**Table 1:** Subject demographics of both CI groups.

Pair	Group	Cause of Deafness	Gender	Hearing Age[years; month]	Age at Implantation [years;month]	Measured CI	CI-speech processor (measured)
1	GP	unknown	male	12;0	3;1	right	Esprit 3G ACE/1200
	BP	unknown	male	13;2	4;4	right	Esprit 3G ACE/900
2	GP	connexin 26	male	10;10	2;1	right	Esprit 3G ACE/1200
	BP	unknown	male	9;3	4;2	left	Freedom ACE/900
3	GP	unknown	male	7;7	3;3	right	Esprit 3G ACE/1200
	BP	unknown	male	5;6	4;5	right	Freedom ACE/1800
4	GP	cytomegaly	female	12;0	3;6	left	Esprit 3G ACE/1200
	BP	cytomegaly; peripartal hypoxia	female	13;1	3;10	right	Esprit 3G ACE/1200
5	GP	unknown	male	15;3	2;0	right	Esprit 3G SPEAK/250
	BP	unknown	male	14;9	3;3	right	Freedom SPEAK/250
6	GP	unknown	female	12	3;0	right	Freedom ACE/1200
	BP	unknown	male	9;2	4;0	right	Freedom ACE/1200
7	GP	unknown	female	6;1	2;0	right	Esprit 3G ACE/1200
	BP	unknown	female	8;2	1;11	right	Freedom ACE/1200
8	GP	unknown	male	6;9	1;2	right	Esprit 3G ACE/1200
	BP	unknown	female	6;4	1;7	right	Freedom ACE/1200
9*	GP	unknown	female	with CI: 2;5; with hearing aid: 19	17;3	right	Freedom ACE/900
	BP	probably by ototoxic antibiotics	female	with CI: 2;3; with hearing aid: 13	13;0	right	Freedom ACE/900
		Mean (SD):		10;9 (3;8)	4;4 (4;1)		
		Mean (SD) without pair 9:		10;2 (3;2)	2;11 (1;1)		

*Demographic Data and CI-specific Information in Both Patient Groups based on their Medical Records.* Matched partners (GP: good performers; BP: bad performers) presented in successive rows. Pairs were matched according to hearing age and age at implantation. Note that hearing experience has a lesser influence on speech performance with increasing age.

Eleven CI users had bilateral and seven had unilateral cochlear implants. All CI users except for one pair that was implanted at the University Hospital Hannover were patients of the Department of Phoniatrics and Pedaudiology of the Muenster University Hospital, Germany. As a consequence, group members received the same type of rehabilitational treatment procedures.

All participants were provided with CIs from Cochlear® (Cochlear, Sydney, Australia), with nine children using *Freedom* speech processors (bad performers = 7; good performers = 2) and nine using an *Esprit 3G* (bad performers = 2; good performers = 7). Note that good performers mainly used the *Esprit 3G* speech processor, the precursor of the *Freedom* processor, when visiting our laboratory. Bad performers in contrary mainly used the *Freedom* processor as a follow up to wearing the *Esprit 3G*. This ultimately unsuccessful change was initiated by physicians and parents, who had hoped for an additional gain in speech performance due to a change in processor technology. Parents whose children developed well preferred not to change the processors. For further information about CI-related processing strategies and participant data, see Table 1.

To equalize auditory processing in participants with one or two cochlear implants, those that were bilaterally provided were measured only with their first implanted CI. Therefore, the right ear was assessed in 16 individuals and the left ear in two individuals. Although no hearing ability remained in the contralateral ear, all participants received an earplug.

#### Healthy controls

To determine phoneme discrimination abilities and the MMN in children with normal hearing, an aged-matched control group (4 boys and 5 girls; 8 to 20 years, mean = 14 years) was tested. The same ear was stimulated in controls as in their matched partners and the other ear was closed using an earplug. All participants received 10€ per hour.

#### Ethics statement

All participants (or their parents in case children were younger than 18 years) provided their written informed consent to participate in this study. The study was approved by the Ethical Committee of the Deutsche Gesellschaft für Psychologie (DGPS) in conformance with the 2004 declaration of Helsinki.

#### General procedure

After 1.5 hours of logopedic and phoniatric assessment by our speech therapist, phoneme discrimination abilities and auditory working memory functions were assessed in all participants. The EEG was then measured in a camera silens, a soundproof and electrically shielded chamber. Subjects watched a silent movie of their choice during EEG registration. The whole procedure, including a lunch break and several small breaks, took five to six hours per participant.

### Data Assessment

#### Stimuli and stimulus presentation

Six German phonemes, embedded in syllables, were chosen as stimulus material: /bu/, /bo/, /ba/, /pu/, /be/, /bi/ (all with tense vowels). Stimuli were recorded with Audacity 1.3.beta® using a sample rate of 48,000 Hz and a 16-bit resolution. Stimuli were spoken by a male person and processed using the software Cool Edit Pro 1.2a®. First, stimuli were cut in order to be as equal in length as possible but still sound like the intended syllable. The onset of the sound files was established by selecting a starting point that sounded the most natural. This way, syllables did not start immediately but were carefully faded in to avoid sound artifacts, which resulted in a brief “silence“ (depending on the stimulus, up to 50 ms). Next, stimuli were normalized to 95% of maximum amplitude to avoid clipping and equalized in average RMS values. The syllables ranged from 420 to 451 ms (Ø436 ms) in duration. Stimuli were presented from two loudspeakers placed at an angle of ±20° azimuth approximately 1.5 m in front of the participant, who was seated in a comfortable chair. Presentation 13.0® was used for stimulus delivery (Neurobehavioral Systems, California, USA). To ensure adequate und comparable stimulation levels between participants, loudness was adjusted by presenting the syllable/bo/at different degrees of loudness via an audiometer (Medimate 622D from Otometrics, Taastrup, Denmark) until reliably could be rated as “comfortably loud” using an analog visual scale. As during subsequent MMN and phoneme discrimination testing, all participants had one ear closed with an earplug.

#### Behavioral data and ratings

**Figure 1:**
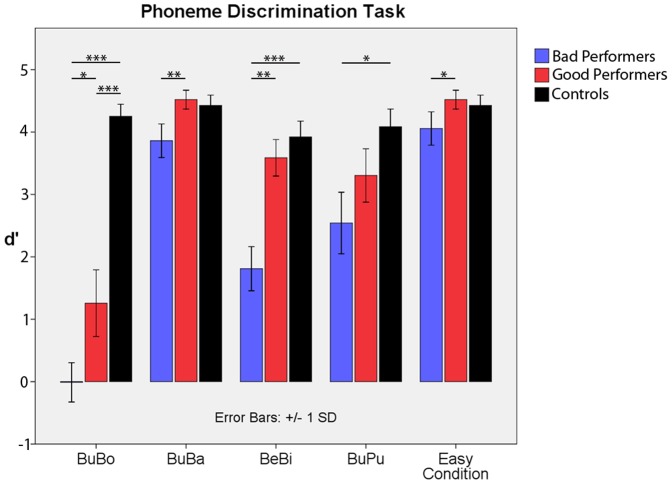
Phoneme Discrimination Task. d’ values for each phoneme discrimination test for each of the groups and the averaged easy condition individually chosen for each participant. A paired t-test confirmed our hypotheses that (1): good performers were significantly better in phoneme discrimination than bad performers (3 out of 4 subtests); (2): the good performer would score equally high compared to healthy controls (3 out of 4 subtests); and (3) that bad performers would show lower performance than controls (3 out of 4 subtests). This was indicated by t-values ranging from 1.7 to 15.21 and p-values ranging from 0.05 (marked by an asterisk) to ≤0.01 (marked by two or more asterisks).

1. Phoneme Discrimination Task: To assess phoneme discrimination abilities, four stimulus pairs varying in phonological similarity were presented: /bu/ vs. /bo/, /bu/ vs. /ba/, /bu/ vs. /pu/ and /be/vs. /bi/(all with tense vowels)./bu/thus appeared in three of four pairs. This was done in order to identify the easiest pair for each individual CI user to be used for subsequent MMN assessment, where/bu/served as the target stimulus for all subjects. /be/ vs. /bi/ was used to extend phonological discrimination beyond the vowel /u/. All participants were asked to differentiate between stimulus pairs in a forced-choice design by deciding via mouse click which stimulus had just been presented. The test order was randomized across participants and each test consisted of 60 repetitions, with each of the two syllables being equally likely to appear. We assessed for each of the subtests hits, misses, false alarms and correct rejections to calculate the sensitivity index d’ (see Fig. 1).

2. Auditory Working Memory: To measure auditory sensory memory, an auditory digit span test was administered and the total number of digits that could be recalled from a given target list was registered. The examiner presented the test items while facing the child. We opted for a forward digit span test, a subtest of the *Psycholinguistischer Entwicklungstest* (PET, [69], the German adaptation of the *Illinois Test of Psycholinguistic Abilities* [70]). The forward digit span of the PET was chosen since it had been intensively validated and is age-group-standardized. It correlates with the non-word repetition test [21], another widely used test for assessing auditory working memory functions. In contrast to other tests (e.g., the backward digit span test; [71]), the forward digit span test relies less on central-executive performance and thereby mirrors very basic levels of auditory working memory close to auditory sensory memory. The forward digit span test had also been used in earlier studies to demonstrate the relation of auditory working memory functions and the MMN [24], [72], which is a measure of auditory sensory memory [5]. In one child, assessment of forward digit span could not be completed.

3. Subjective Satisfaction Rating: All CI users were asked to rate their personal satisfaction with their speech perception (12 items). This rating focused mainly on daily life situations, for example, “*How often do you talk to somebody on the phone if you don’t know this person?”.* Items were ordinally scaled (5-point rating scale) using the levels: “never”, “rarely ever”, “sometimes”, “often” and “always”. The sum over twelve items thus ranged from twelve to 60 points. Items were adapted from the *Manchester Teen Questionnaire* part of the *TeenEars Testbox* [68] (see Table S2).

#### EEG data recording

Mismatch Negativity was assessed in the EEG by using an odd-ball paradigm with a ratio of 85∶15 (standard:deviant in percent), resulting in 803 standards and 141 deviants per run. To account for individual differences due to the implant, the easiest of three pairs (/bu/ vs. /ba/, /bu/ vs. /bo/, bu/ vs. /pu/) was identified for each CI user and used as stimulus material in EEG registration. In 17 CI users/bu/vs./ba/was chosen and in one bu/ vs. /pu/. All healthy controls were stimulated with /bu/ vs. /ba/, the most dissimilar – and therefore easiest to differentiate – pair. Note that /bu/ was presented to all participants, with the advantage that /bu/ can be used as standard (run 1) and as deviant (run 2) during EEG measurement. With /bu/ both as deviant and standard, the resulting MMN is argued to be free of pure stimulus differences [73]. The artifact produced by the cochlear implant is also identical for deviant and standard with this design and can be eliminated by subtracting standard from deviant, as has already been successfully demonstrated [74].

With an average stimulus length of 436 ms and an inter-stimulus interval of 900 ms (with a jittering of ±200 ms), each of the two runs lasted about 20 minutes. To measure brain responses, the hardware and software of the Brain Products system was used (www.brainproducts.com). A 32-channel EEG cap (model “Easycap BrainCap-MR 3-0 32Ch) with electrodes located according to the international 10–20 system was employed. The placement of the 32 electrodes covered evenly an area as large as possible especially over inferior fronto-temporal and inferior occipital regions. This arrangement of electrodes is especially useful if average reference is employed. During measurement, electrode impedances were kept below 5kΩ and FCz was used as the reference channel. Eye blinks were recorded with an additional EOG electrode, which was centered below the right eye. Electrodes that were located above the cochlear implant were not prepared for measurement. This involved on average 2.8 electrodes per subject and has been shown not to influence source localization in CI users [64], [66]. Data were passed to the amplifier (Brain Amp MR, 32 channels), where they were filtered online with 0.1 Hz–250 Hz and recorded with a sampling rate of 500 Hz using Brain Vision Recorder®. To account for interindividual differences, CI users’ individual electrode positions were digitized with Polhemus Fastrack® 3D.

### Data Processing and Analyses

#### Behavioral data and ratings

1. Phoneme Discrimination Task: The sensitivity index d’ was individually calculated for each stimulus pair. A discriminant analysis was performed to assess whether individual performance in phoneme discrimination could correctly predict group membership. A repeated measures ANOVA was calculated to identify differences between groups. Paired t-tests were used to investigate effects more in detail [75].

2. Auditory Working Memory: Differences between CI groups were tested using paired-t tests. To account for the years of deafness, differences were not only tested by standardizing test values according to age (years of life) but also to each patient’s hearing age (by taking the duration of hearing experience instead of age for standardization).

#### EEG data analysis

**Figure 2:**
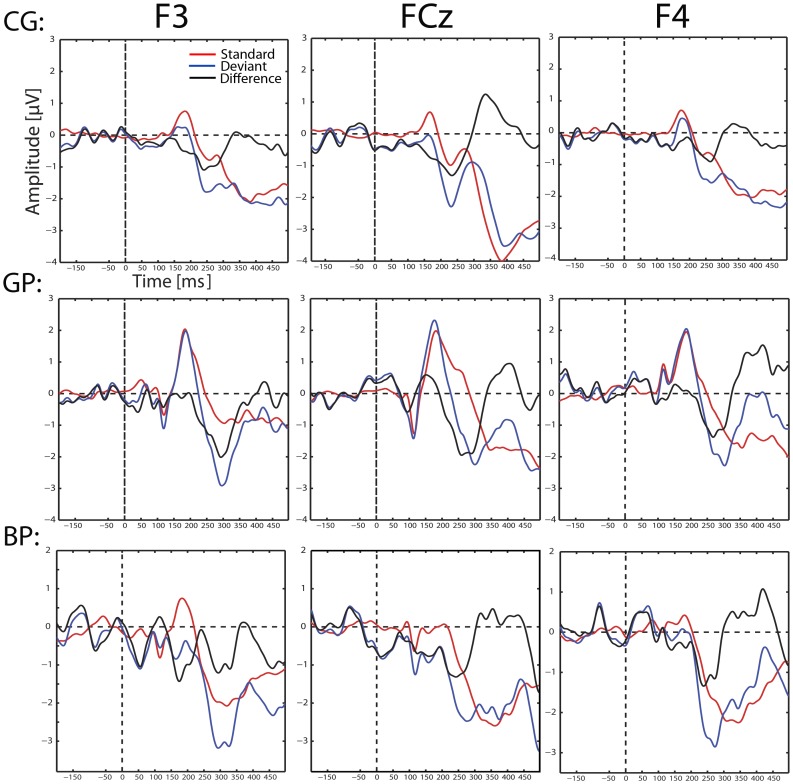
The MMN in Sensor Space. Standards (red), deviants (blue) and their difference waveform (black) are shown at central (FCz) and frontal (F3 and F4) positions for all three groups to facilitate comparison of the later described effects in source space. Average reference was used. The MMN appeared in sensor space for the control group from 220 to 310 ms (120–210 ms lc) and for CI users from 230 to 350 ms (130 to 250 ms lc). As in source space, the MMN seems to be stronger in the good performers than in the bad performers. The double peak of the MMN found in the bad performers in source space can also be seen here (F3, F4). The CI artifact is visible in all CI users at 114 ms (14 ms lc).

To analyze the Mismatch Negativity, EEG data were imported to BESA 5.3. To account for interindividual differences, each CI user’s electrode positions were normalized and dead channels were interpolated. Because patient EEG data are often of bad quality, rejection of eye-blink distorted trials would have severely reduced the number of trials. Therefore, blink activity was corrected in every CI user by using the adaptive artifact correction method provided by BESA® [76]. This artifact correction method is based on the eye blink topography that is only estimated from surface electrodes and not polygraphic channels (i.e., eye electrodes). The eye electrode below the eye was simply used to facilitate the pattern search algorithm, which detects eye blinks. All detected eye blinks were averaged and the average eye blink was then decomposed into principle components. PCA analysis was solely based on surface channel data. The first PCA component, which usually explains more than 95% of the average eye blink, is then subsequently subtracted from the data. As a consequence, brain activity and artifact are disentangled and the procedure of blink removal does not result in distorted brain waves, especially in frontal EEG channels. Continuous blink-free EEG data were filtered from 0.1 to 25 Hz and epoched from −200 to 500 ms with a baseline correction from −175 to 0 ms (see Fig. 2). Remaining artifacts were removed by applying the artifact scan tool implemented to BESA®. Only those standards that were not preceded by a deviant were averaged. In CI users, an average of 552 standards and 116 deviants remained for further analyses (controls: 624 standards, 130 deviants on average). After averaging, reference was recomputed from FCz to average reference. We chose average reference following the guidelines for using human event-related potentials to study cognition [73]. This reference is suggested to avoid a reference bias especially in the interpretation of topographic differences and for source analyses. Finally, individual electrode positions were transformed into standard positions and data were exported via MATLAB 2009a® to Emegs2.5® [77].

**Figure 3:**
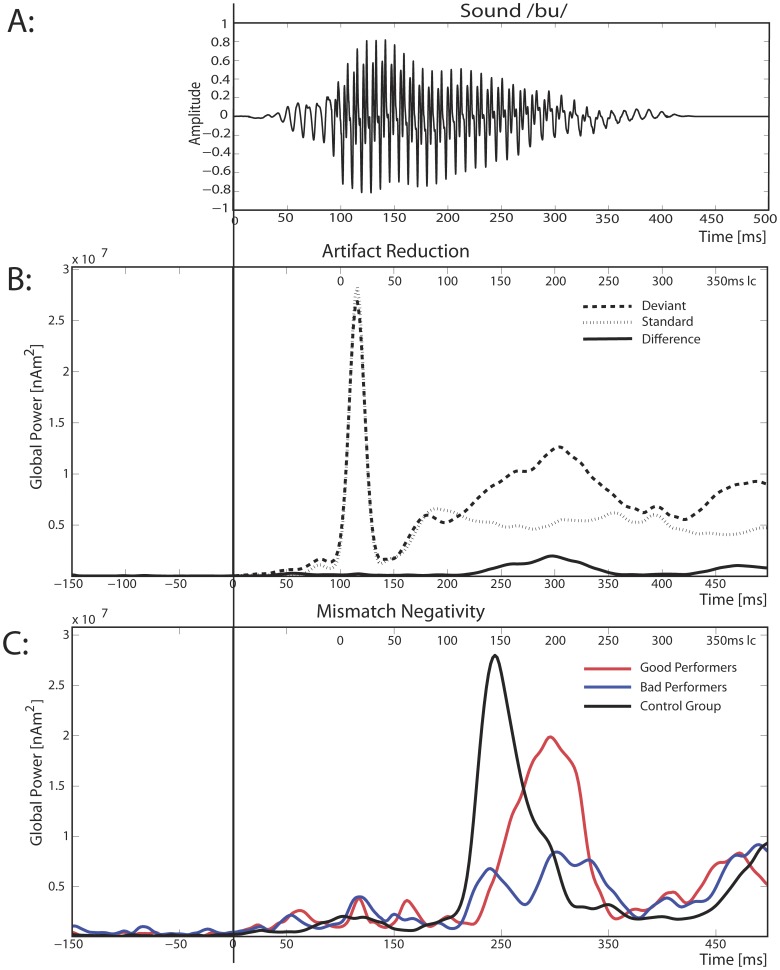
Global Power Plots. **2A:** Sound wave form of the target stimulus /bu/. **2B:** Separate global power of the minimum norm estimates for standards and deviants as well as their difference (MMN). Note that the CI artifact is clearly visible at 114 ms (14 ms lc) in standards and deviants, whereas it is totally diminished in the difference waveform. **2C:** Global power of the minimum norm estimates for each of the three groups.

In Emegs, cortical sources of the event-related fields were separately estimated for standards and deviants using the L2 Minimum-Norm Estimates (L2-MNE) method [59]. The L2-MME is an inverse modeling technique applied to reconstruct the topography of the primary current underlying the electric field distribution. It allows the estimation of distributed neural-network activity without a priori assumptions regarding the location and/or number of current sources [60]. In addition, of all possible generator sources, only those exclusively determined by measured electric fields are considered. Calculation of the L2-MNE was based on a spherical four-shell isotropic volume conductor headmodel with 3 (radial, azimuthal, and polar direction) ×127 evenly and spherically distributed dipoles as a source model. A source shell radius of 6 cm was chosen as a trade-off between depth sensitivity and spatial resolution (Tikhonov regularization parameter k = 0.1). Although the distributed source reconstruction in EEG does not give the precise localization of cerebral generators, it allows for an approximation of cortical generators and corresponding assignment to larger cortical structures. To establish the MMN, standards and deviants were averaged for each subject; standards were then subtracted from deviants for each participant. The global power across all dipoles was then plotted over time to identify the time course of the MMN in all three groups, (see Fig. 3C).

For statistical analysis of the two groups of CI users, a point-wise repeated measures ANOVA was calculated for every dipole and time point of the MMN [10], [61]. Note that the difference waveforms of deviants and standards were used for analyses, resulting in only the between-factor “group” (good vs. bad performers). As an outcome of this analysis, a spatiotemporal distribution of statistical values for each dipole and time-point was obtained. To prevent interpretation of random effects, only adjacent dipoles displaying significant F-values (p<0.05) over at least ten consecutive data points for the factor “group” were clustered and then averaged. In line with the literature, two clusters were found in the typical time range of the MMN: cluster 1 consisted of 25 dipoles and was centrally localized in the frontal cortex; cluster 2, consisting of nine dipoles, was localized in the left temporal lobe with a focus on the auditory cortex. For both clusters, Pearson’s correlation tests were computed to identify possible relations between brain activity (defined by its cluster-based averaged source strength) and phoneme discrimination (easy condition), auditory working memory (forward digit span test), global speech performance, each CI user’s satisfaction with their ability to perceive speech and age at implantation.

Due to the rather complex stimulus material used in the present study, latencies of the MMN were expected to be delayed in all groups. As mentioned in the stimulus section, this was caused by a brief period of silence preceding the syllable that was necessary to insert in order to make the stimulus material sound more natural and was approximately 50 ms long. The MMN was also not triggered to the stimulus onset but rather by the syllable perception point, meaning the time point at which standard and deviant could reliably be differentiated [78]. Apart from co-articulary influences, this appeared at the transition from consonant to vowels, emerging roughly at 100 ms, as can be seen in Fig. 3A, where the energy of the signal becomes strongly enhanced. In the results section, both latencies (corrected and uncorrected) will be presented. In the discussion, only corrected latencies (lc) times will be mentioned.

#### Artifacts evoked by the implant

**Figure 4:**
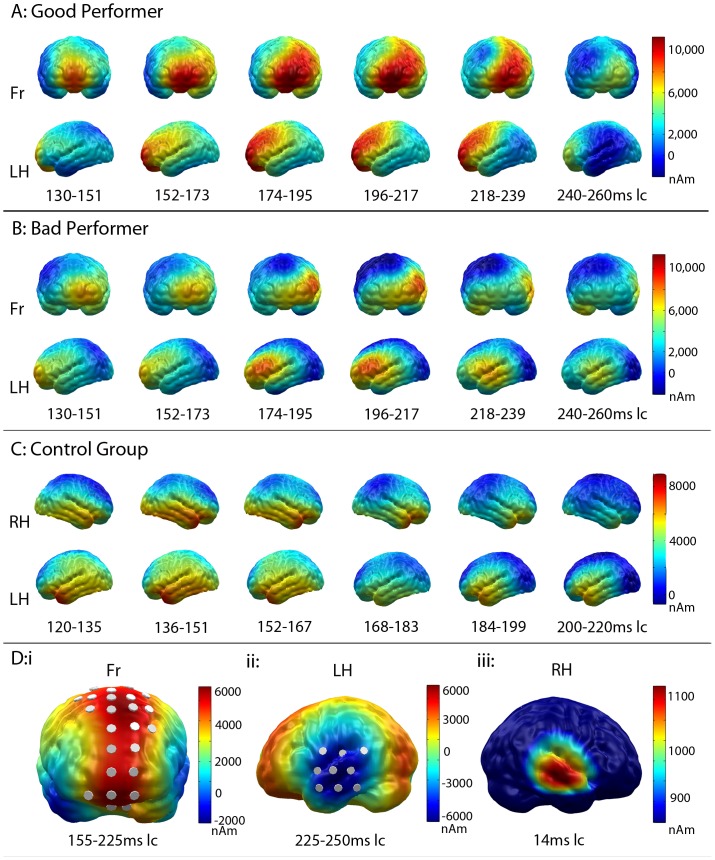
MMN Source Localization and Group Differences. Source localization of MMN in good performers (**4A**), bad performers (**4B**) and controls (**4C**): in both patient groups, the left frontal cortex was activated from 130 to 240 ms lc. In bad performers, the auditory cortex was additionally involved (218–260 ms lc). Healthy controls displayed bilateral activity in fronto-temporal regions from 120 to 210 ms lc. **4Di/ii:** Difference plots of the minimum norm estimates of good minus bad performers during the early (**Di**) and late (**Dii**) interval of the MMN. Grey discs indicate adjacent dipole locations within the two clusters (cluster 1: frontal; cluster 2: left temporal). While good performers showed significantly more activity in the frontal cortex from 155 to 225 ms lc (**Di**, indicated in red), this was followed by a stronger activation of the auditory cortex in the bad performers from 225 to 250 ms lc (**Dii**, indicated in blue). **Diii:** Correct source localization of the averaged CI artifact in the 16 patients who wore their CI on the right side.

An important issue for ERPs of CI-users is the artifact produced by the cochlear implant. The neural signature of CI artifacts in time is shown separately for standards and deviants in Fig. 3B and its location in Fig. 4Diii. To assess potential influences of the artifact on localization and time course of the MMN, we considered the effect after subtracting standards from deviants, which entirely removed artifactual activity from the ERP. This approach to cope with CI artifacts had already been successfully used [74].

Data from controls were used to display the time course and localization of the MMN in subjects with normal hearing. Therefore, data were analyzed by first subtracting the L2 Minimum-Norm Estimates of the standards from those of the deviants for each subject. Then, difference waveforms were averaged within each group and displayed in a global power plot to obtain the latency of the Mismatch Negativity. Regions of interest were predefined by the literature and selected by visual inspection of the data, namely in the left and right temporal and prefrontal cortex, consisting of 15 dipoles each. Data for both clusters were then exported and analyzed in SPSS. A paired t-test was computed to find hemispheric differences in the MMN and a Pearson’s correlation test was additionally calculated for the strength of the MMN of each hemisphere and phoneme discrimination abilities (easy condition).

All p-values for results were Greenhouse-Geisser corrected for non-sphericity (if necessary). In effects that were not derived from hypotheses, p-values of multiple paired t-tests/multiple correlation tests were bonferroni-corrected.

## Results

### Behavioral Data and Ratings

#### 1. Phoneme discrimination task

The repeated measures ANOVA with the factor “group” (comparing controls, good and bad performers) and “phoneme subtest” (comparing the four phoneme pairs) revealed a significant main effect “group” (F_(4,32)_ = 46.9, p<0.001), “phoneme subtest” (F_(2,16)_ = 35.9, p<0.001) and a significant interaction of both (F_(8,64)_ = 7.62, p<0.001). As expected, participants in the control group showed excellent phoneme discrimination abilities in all subtests, with an average d’ of 4.16 (which equals about 98% correct answers). Post-hoc t-tests revealed that the good CI performers showed phonological discrimination very similar to controls (average d’ = 3.16, 87% correct answers). Control subjects significantly outperformed the good performers in only one subtest (/bu/ vs. /bo/). In contrast, results from good and bad performers significantly differed, with an average d’ of 2.05 (76% correct answers) for bad performers. This difference was especially strong in /be/ vs. /bi/ and /bu/ vs. /ba/ and weaker (but still significant) in /bu/ vs. /bo/. Only one subtest (/bu/ vs. /pu/) did not reveal a significant difference between groups. For comparison and statistical significance, see Fig. 1. Sorted according to each individual’s easiest subtest, it is clear that all groups responded at a high performance level, but with a significantly better performance in the good than in the bad performers. These results emphasize the validity of phoneme discrimination ability for distinguishing between good and bad performers at a group level.

To investigate its predictive value on speech performance at a single-case level, we calculated a discriminant analysis. Of all analyzed variables (d’ of /bu/ vs. /bo/, /bu/ vs. /ba/, /be/ vs. /bi/, /bu/ vs./pu/, age at implantation), two phoneme discrimination subtests went into the model (entry criterion: F = 3.84, remove criterion: F = 2.71) in the following order: /be/ vs. /bi/ (F = 20.92 including pair 9/F = 13.95 without pair 9) and /bu/ vs. /ba/ (F = 8.84 including pair 9/F = 8.03 without pair 9). Age at implantation did not reach significance as a predictor variable (F = 0.033 including pair 9/F = 0.156 without pair 9). Based on these two pairs alone, the probability of belonging to the predicted group given these discriminant scores was on average 100% for bad performers and 88.9% for good performers. Group membership of 17/18 CI users (95%) could thus be correctly predicted, confirming the classification established on a large, time-consuming test battery at a single-case level.

#### 2. Working Memory Functions

On average, CI users reached a percentile rank of 21.5 in the test norms of the PET (based on chronological age, std = 21.9) or 41.4 (based on their hearing age, std = 11.39). Compared to a percentile rank of 50, CI users were significantly worse than the average population (t_(16)_ = 5.4, p<0.001 based on chronological age; t_(16)_ = 3.1, p<0.01 based on hearing age). No significant difference could be found between the two CI groups (t_(7)_ = 0.96, p = 0.18 including pair 9, t_(6)_ = 1.48, p = 0.09 without pair 9, both comparisons based on hearing age).

### EEG Data

For comparing the MMN to results of previous studies, standards, deviants and their difference waveforms are displayed for all three groups in sensor space at three locations (F3, FCz, F4) in Fig. 2.

Fig. 3B separately displays the global power of the L2-MNE for deviants, standards and their difference averaged for all CI users. Standards and deviants both clearly showed the impact of the artifact caused by the implant. As expected, the artifact was identical in deviants and standards and appeared between 100 and 130 ms (0–30 ms latency corrected (lc)) with a maximum at 114 ms (14 ms lc). As a consequence, it completely disappeared after calculating the standard-deviant difference. Fig. 4Diii shows the artifact localization in the 16 CI users who wore their implants on the right side. As expected, it was correctly located in the right temporal lobe, not confounding the MMN results, as is shown in the following.

Inspecting the time course of the MMN of the control participants, a clear activity from 220 to 310 ms (120–210 ms lc) was found (see Fig. 3C). Projecting the L2-MNE source solutions onto a cortical surface demonstrated a bilateral activation of fronto-temporal regions (Fig. 4C) that showed no hemispheric dominance (t_(8)_ = 0.169, p = 0.87). No correlation was found between phoneme discrimination of the easy condition and the source strength of the MMN (left: r = −0.014, p = 0.97; right: r = −0.046, p = 0.91).

The difference waves of CI users in Fig. 3B revealed an MMN from 230 to 350 ms (130–250 ms lc). In Fig. 3C, the MMN of the good performers is clearly visible, while that of the bad performers showed a strongly attenuated amplitude.

Fig. 4 A–C shows the L2-MNE separately projected onto a cortical surface for all groups. Visual inspection suggested that the MMN was most strongly expressed in left frontal areas in the good performers. Left temporal regions were also involved, albeit to a lesser degree. Left frontal activation was also observed in bad performers, but was less prominent than in good performers. Instead, they additionally showed an activation of the left temporal cortex, especially in regions associated with auditory analyses. These visual impressions were confirmed by statistical analyses. In a point-wise repeated measures ANOVA with the factor “group” based on the difference between standards and deviants, two time windows reached the predefined significance threshold: an earlier one between 255 and 325 ms (155 - 225 ms lc) and a later one between 325 and 350 ms (225–250 ms lc).

In the earlier interval, a difference between groups was seen in the central frontal cortex (averaging 25 dipoles for cluster 1 from 255–325 ms (155–225 ms lc); F_(1,16)_ = 5.6, p = 0.031), with higher activity in the good than in the bad performers. This effect and the corresponding dipoles are illustrated in Fig. 4Di, which shows the difference plot of good (mean = 6468 nAm, std = 1657) vs. bad performers (mean = 1531 nAm, std = 1261).

This effect was followed by a stronger activation of the left temporal cortex in the bad performers (averaging 9 dipoles for cluster 2 from 325–350 ms (225–250 ms lc); F_(1,16)_ = 10.7, p = 0.005). Fig. 4Dii displays this difference between good (mean = −586 nAm, std = 2258) and bad performers (mean = 5111 nAm, std = 4705). During the first interval, no significant difference was observed between groups in temporal areas (Cluster 2) and no significant group effect was found in central frontal areas during the latter time window (Cluster 1).

**Figure 5:**
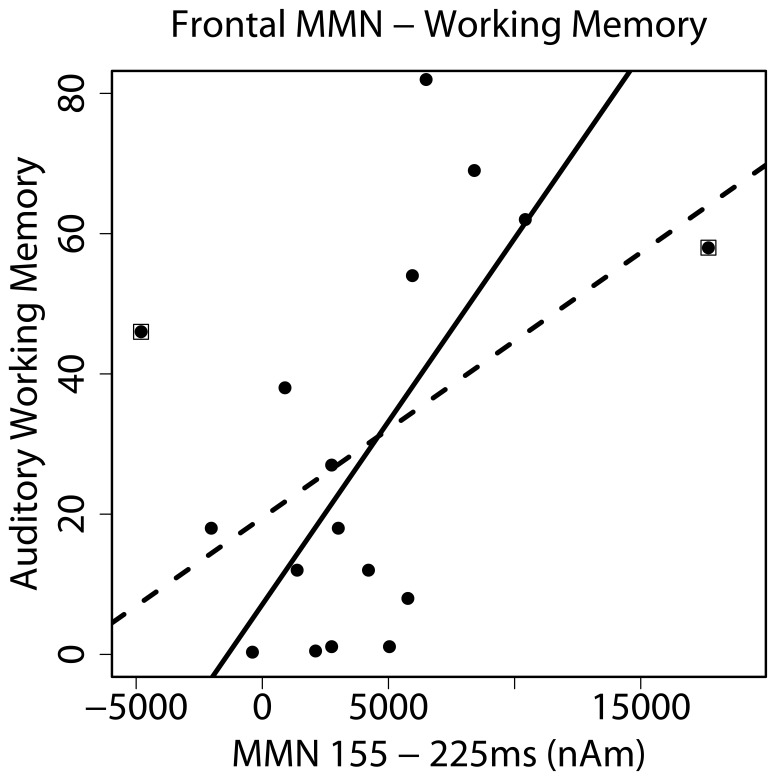
Influence of Frontal Activation on Auditory Working Memory. Correlation of the central frontal cortex, in which good performers showed significantly higher activity than bad performers, with auditory working memory (dashed line). Correlation strength increased after the removal of two outliers (straight line, outliers marked with squares), indicating that stronger frontal activation was connected to better auditory working memory.

In the following, we establish the relation of the strength of the MMN to behavioral measures. A significant correlation between the neural activity of the central frontal cluster and the digit span test adjusted for hearing age (r = 0.48, p = 0.05, N = 17, Fig. 5 dashed line) was obtained, indicating that CI users with higher frontal activity also displayed better auditory working memory (the significant correlation remained even after removing two outliers from the analysis (r = 0.63, p = 0.01, N = 15, straight line) and also using Spearman’s rank correlation test to control for abnormally distributed data (r = 0.52, p = 0.03, N = 17).

There was also a highly significant correlation between global speech performance and frontal activation, indicating that a stronger activation of the frontal cortex corresponded with better global speech performance in CI users (r = −0.591, p = 0.01, N = 18). The frontal activity also displayed a trend for a positive correlation with the subjective satisfaction with speech perception (r = 0.45, p = 0.06, N = 18).

For the latter MMN interval, there was a significant correlation between the temporal cortex and global speech performance, revealing that stronger activation of the temporal cortex from 325 to 350 ms (225–250 ms lc) was associated with a lower global speech performance in CI users (r = 0.54, p = 0.02, N = 18). There was also a trend towards a negative correlation with the ability to discriminate the phonemes measured in the EEG recording (r = −0.45, p = 0.06, N = 18), indicating that a stronger activation of the temporal cortex corresponded with *weaker* phoneme discrimination abilities. The significant correlations found between behavioral measures and the strength of the MMN further underline the group differences found in the early and late MMN components.

No other correlations between brain activity and behavioral measures reached standard criteria of significance (most importantly for age at implantation: MMN frontal cortex: r = −0.26, p = 0.29 including pair 9, r = −0.28, p = 0.29 without pair 9; MMN temporal cortex: r = 0.2, p = 0.42 including 9, r = 0.18, p = 0.5 without pair 9).

## Discussion

The results of this study underline the importance of phoneme discrimination and auditory sensory memory for speech development in prelingually deafened cochlear implant recipients. Phoneme discrimination and memory functions were assessed by behavioral and electrophysiological (MMN) means in children with very good and very poor speech performance, who were matched according to hearing experience and age at implantation. Moreover, the investigation of cortical generators of the MMN provided new insights into group-specific cortical speech processing strategies and stressed the role of the prefrontal cortex in the positive development of speech following implantation. Additionally, an age-matched control group with normal hearing was assessed in which phoneme discrimination was measured in behavior and EEG.

Although a prerequisite for the selection of participants was very good hearing ability (as indexed by the ability to repeat monosyllabic words and to carry out telephone calls), good performers behaviorally displayed a higher performance in these abilities than bad performers. However, regarding the ability to discriminate phonemes, the differences between these two groups became drastic, with good performers even equaling the performance of controls in almost every subtest. This was further emphasized by means of a discriminant analyses that enabled the correct prediction of individual group membership (good vs. bad performers) in all but one of the CI users with no more than two phoneme discrimination tests. Differences in the auditory memory between groups were not seen in the behavioral test, but by means of the MMN.

In healthy controls, the MMN was bilaterally identified from 120 to 210 ms in temporal and prefrontal areas, showing no correlation with phoneme discrimination. In CI users, it appeared from 130 to 250 ms in fronto-temporal regions of the left hemisphere. Comparing the good and bad performers revealed that it was divided into two parts: in the earlier time window from 155 to 225 ms, good performers showed a strong activation of the central frontal cortex that was followed from 225 to 250 ms by increased activity in the left temporal lobe seen only in the bad performers. The strength of the frontal activation was positively correlated with the behavioral working memory test, global speech performance and (as a trend) with each person’s satisfaction with their own speech perception. In contrast, the activation strength of the temporal cortex showed a negative correlation with global speech performance and (as a trend) with phoneme discrimination. Neither frontal activation correlated with phonological awareness nor the activation of the temporal cortex with the behavioral auditory working memory test. The following discussion addresses the localization of the MMN, its time course and underlying processing strategies.

### MMN Localization

In CI users, the MMN was found from 130 to 250 ms in the left hemisphere, spanning from frontal to temporal regions and supporting previous reports of the neuroimaging literature [50], [79]–[81]. Of course, we acknowledge that techniques such as PET or fMRI would have offered better MMN localization, but they would have been of limited use to study the MMN in CI users and particularly in children because of the invasive character, strong safety concerns and, in general, lower temporal resolution [82].

While the MMN was distributed equally strong across both hemispheres in earlier studies as well as in the healthy controls displayed here, a rather left-hemispheric distribution was found in both CI groups. This may seem to be an unexpected result, but there are several potential explanations for such a finding. It has been extensively shown that the auditory system displays hemispheric specialization. While the right hemisphere is more sensitive to spectral information, the left hemisphere focuses on rapidly changing acoustic cues provided by speech stimuli (for a review: [83]). Moreover, stimulation was unilaterally presented to the first implanted ear in CI users (in 16/18 cases, the right ear), which was likely to be followed by enhanced activity of the contralateral hemisphere [84]–[86].

Finally, due to technical limitations, cochlear implants do not provide detailed spectral and temporal information [87]. Processing of complex sounds as provided by speech and music is thus limited and cochlear implant users have to develop perceptual strategies to interpret the incoming information. Complex sounds can be analyzed via their spectral or fundamental frequencies, with spectral frequency being processed in the right and fundamental frequency in the left hemisphere [88]. It has already been discussed that CI users rely on fundamental frequencies to interpret complex sounds [66], [87]. Therefore, the left hemisphere should mainly be active in these individuals, while – as also shown in our experiment – both hemispheres are activated in controls with normal hearing.

### Time Course of the MMN

The MMN reported in our study shows a rather wide time interval from 130 to 250 ms post-onset in CI-users, which was probably caused by two factors: first, in the present study, children and young adults aged seven to 19 were tested. It has been repeatedly shown that ERP latency decreases with increasing age [89], [90], which in turn broadens the averaged MMN, as seen here. Second, phonemes trigger a rather wide MMN [40], [91], [92] in children and in adults [93]. Our results are therefore in agreement with the literature. Still there is another possibility that could trigger a rather broad MMN. In the current design, a deviant-minus-physically identical standard ERP-paradigm was used to trigger the MMN. Although this is a very progressive approach, it still does not account for refractory processes that decrease the N1 in standards compared to deviants [94]. Especially in cases, where the N1 and the MMN superimpose, this can lead to group differences that are not fully caused by the MMN but additionally by N1-refractoriness in the standards (which may broaden the MMN). This problem could be controlled in future studies by designing a new control condition (run 2) in which the standards and deviants are not simply exchanged, but the deviant is presented within other stimuli having the same absolute number of occurrence, but showing no predictable patterns as standards and deviants in run 1 [95].

As mentioned above, for each CI user, the most easily differentiable phoneme pair of the four pairs presented in the phoneme discrimination task was chosen for EEG stimulation. In 17 out of 18 CI users, this was/bu/vs./ba/and/bu/vs./pu/in only one user. The MMN is triggered by the syllable perception point, meaning the time point at which standard and deviant can be reliably differentiated [78]. Thus, in /bu/ vs. /pu/, this point is reached considerably more early than in /bu/ vs. /ba/ due to the different consonants at the onset of /bu/ vs. /pu/. As a consequence, it is likely that the MMN possibly appeared earlier in one CI user (who belonged to the bad performers) than in all other CI users and the control group. This added additional variance to the MMN latency of the bad performers and might have weakened the statistical effects found in the current study.

### Speech Processing Strategies in Good and Bad Performers

As hypothesized above, we expected all three groups to show frontal and temporal sources for the MMN, reflecting possible group differences in auditory sensory memory and phoneme discrimination. Summarizing our results, we consider the most interesting findings first of all that good performers showed strong activity in the frontal cortex, while bad performers (when directly compared) did not to the same extent. Though no significant difference in auditory working memory was found at a behavioral level, the strength of this frontal activity over a time range from 155 to 225 ms was positively correlated with the forward digit span test, with better auditory working memory being reflected in stronger frontal activity. Second, following frontal activation, enhanced activity in the left temporal cortex with a special focus on the auditory cortex was found in the bad performers from 225 to 250 ms. This activation showed a trend towards negative correlation with phoneme discrimination, indicating that the CI users who had the greatest problems with phoneme discrimination recruited their temporal cortices the most strongly.

But which technical, clinical or neurocognitive factors can account for our findings? One explanation could be a better technological equipment that was only provided to the good performers. However, bad performers used the newer speech processor technology (*Freedom*, ratio of users: 7∶2) as a follow-up to the use of the *Esprit 3G* far more often, whereas good performers had reached satisfactory hearing with the *Esprit 3G* alone (ratio of users: 7∶2). Therefore, better speech processors provide no likely explanation for better speech development. Having received the same treatment conditions and rehabilitation programs in our clinic from the onset, systematic differences in CI surgery and post-rehabilitational support also do not seem to be a likely explanation.

Another possible explanation for group differences could be the lower age at implantation of the good performers. Although we tried to match CI groups according to hearing age and age at implantation as well as possible, good performers were implanted on average eleven months earlier than bad performers (at least when pair 9 was excluded from group comparisons). It is therefore possible that age at implantation influenced cortical plasticity after CI implantation. Still, we used age at implantation as an additional variable in all subsequent analyses, which had no influence on our main findings: with a hit rate of 95%, phoneme discrimination abilities were a very powerful predictor for group membership, while age at implantation did not account for more variance. Also, while phoneme discrimination and the behavioral auditory working memory test were related to brain activity, this was not so for age at implantation.

Group differences could be also overestimated due to a negative shift in sensor space seen at FCz in the bad performers and the control group. These shift appears from −50 ms to app. 80 ms, covering parts of the baseline (−50–0 ms). At the same electrode and time range but with a reversed polarity, a positive shift is found in the good performers. It is possible, that these shifts influence the strength of the MMN in all three groups. This means, that the negative shift increases the MMN of the bad performers and the control group, while the positive shift weakens the MMN of the good performers. This would not only affect the ERPs in sensor- but also in source space. To minimize a possible influence on the group difference found in the current study, one could shorten the baseline by 50 ms (−150 to −50 ms). Still, this would result in a weakening of the MMN in the bad performers and the control group and in an increased amplitude in the MMN of the good performers. As a consequence, the group difference found in the current study would become even stronger, which means that the group effect was rather underestimated than the opposite.

Additionally, in the current study the difference waveform (deviant minus standard) was analyzed to find group differences between good and bad performers. Although this is a valid strategy and very often used in MMN studies it does not offer the possibility to test whether differences between groups are based on differences in response to deviants (as the subtraction procedure suggests), to standards or to both. As one can see in Fig. 2, standards in good and bad performers look different. Therefore this could influence the group difference found in the current study. Still, when taking a closer look at standards and deviants, it seems that more general group specific differences in auditory processing exist in both waveforms and become subtracted by calculating the difference waveform. Still this does not necessarily have to be always the case and therefore analyzing standards and deviants separately could be a promising approach for future studies.

Unless other unknown reasons were responsible for the different outcomes in both groups, we favor one of the following explanations for their differences. Given the significantly stronger activation of the frontal cortex in good performers, indicating substantial involvement of auditory working memory [96], [97], it seems plausible that good performers relied more on the auditory sensory memory to encode subtle phonological differences between speech items than bad performers. Bad performers, in contrast, relied more on auditory processing of the incoming input *after* reduced processing in the frontal cortex. It is likely that this temporal activation during MMN displays a compensatory strategy, with the goal to deal best with the sensory signal by enhanced auditory analyses. This is not only supported by the timeline of activations but also by the negative correlation found with phoneme discrimination: sensory activation is normally found in early stages of stimulus analyses (e.g., as displayed in N1) and its amplitude has been shown to mirror successful stimulus processing [98]. Therefore, it would be more plausible to find the temporal activation *preceding* the frontal component in the MMN. Instead, it was not only *subsequent* to the frontal activation, but also more strongly developed in bad performers, correlating *negatively* with phoneme discrimination abilities. It is thus plausible that this late temporal component reflects a compensational strategy – an attempt to reanalyze the auditory input in the relevant sensory cortex – to extract more information.

While this explanation implies a resource-dependent approach to speech encoding, another possibility seems to be that a reduced auditory sensory memory capacity constitutes a specific vulnerability factor. If this holds true, bad performers were *forced* to focus more on reanalyzing auditory input. Still, at a behavioral level, a group difference in auditory working memory was only found as a trend when pair 9 was excluded. With an additional missing data set in one patient, the power of the pair-based group comparison was reduced and interpretation of the statistical trend was thus avoided here. In addition, although the forward digit span test has been shown to be related to auditory sensory memory (by means of the MMN, [24], [72]) it only indirectly allows for measurement of the same. In case reduced auditory sensory memory capacity constitutes a specific vulnerability factor, the targeted effect should thereby be found in future studies with a more direct measure of auditory sensory memory or with a higher number of participants.

In addition to sensory auditory memory functions, attention-related processes could also trigger differences in speech perception and development. As pointed out by Pulvermüller and Shtyrov [40], the MMN is *independent* of attention by persisting even under hardest distraction [34], but it is *dependent* on the attention directed to the presented stimuli [99]–[101]. That means that although it was not required in the current study to pay attention to the presented phonemes, good performers could have identified the deviant stimuli as special and as a consequence switched attention to these stimuli, a process well known to involve the frontal cortex [102]. This hypothesis is further supported by a P3a-like component which is related to the engagement of attention [103] and is clearly seen at FCz for the good performers and the control group, but less obvious for the bad performers. Therefore, this could imply that such a process can be regarded as a vulnerability factor for the successful development of speech.

Finally, the interplay of attention with sensory memory processes could also explain our results. It has been extensively shown that auditory sensory memory is positively influenced by attention in terms of top-down modulation. This was shown in several studies investigating the effects of attention on the early processing levels of working memory functions, including expectancy [104], encoding [105]–[107] and maintenance [108], [109]. Therefore, it could well be that early auditory memory functions were enhanced in good performers by top-down processing, resulting in better speech performance.

Given the current literature, no definite explanation can be offered. To further investigate the mechanisms, we believe that it is necessary to intensively train bad performers in phoneme discrimination and/or auditory working memory in order to see whether frontal activity increases and/or activity in the auditory cortex decreases with increasing performance. Such training could improve all levels of auditory working memory, including auditory sensory memory. It has been already shown that the training of working memory works and phonological awareness is successful and manageable, even when done at home and on a PC [9], [110], [111]. Further exploration of the influence of phoneme discrimination and auditory sensory memory on speech development in CI users, especially by training very young children, thus seems to be a very promising approach.

In conclusion, our results further underline the impact of phonological awareness and auditory sensory memory on speech development. Together with the MMN as an objective and time-sensitive indicator for both functions, standardized assessment of basic auditory memory functions and phonological awareness in clinical routines could provide knowledge about deficits at early stages and could also have a predictive value for later speech development. Training these functions from early on could therefore prevent deficits in later speech performance.

## Supporting Information

Table S1 Rating of Speech Performance.
(DOCX)

Table S2 Subjective Rating of Hearing.
(DOCX)
