# Supplementary material for: Neural Correlates of Speech Processing in Prelingually Deafened Children and Adolescents with Cochlear Implants
Source: PLoS One. 2013 Jul 4;8(7):e67696. doi: 10.1371/journal.pone.0067696 (PMC3701579; doi:10.1371/journal.pone.0067696)
Supplement: Table S1 — Rating of Speech Performance. (DOCX) [file pone.0067696.s001.docx]

|  | **Good Performers** | |  | **Bad Performers** | | |
| --- | --- | --- | --- | --- | --- | --- |
| **Assessment of speech performance** | **1** | **2** | **3** | **4** | **5** | **6** |
| Articulation (based on Fox, 2003) | No conspicuousness; inconspicuous sound of voice in spontaneous speech (no noticeable nasality; prosody and pitch of speaking voice inconspicuous) | Minor phonological abnormalities (e.g. elisions of unstressed syllables); good intelligibility and inconspicuous sound of voice | Mildly impaired comprehensibility, phonological abnormalities (substitution of vowels or consonants; assimilations of consonants; reduction of syllables within words, elisions or addition of consonants) appear repeatedly and/or mild phonetic abnormalities (e.g. dyslalia) | Phonological errors appear frequently and/or 2-3 consonants cannot be formed and/or impaired vocal sound; comprehensibility is reduced | Incomplete phonemic inventory (conspicuous phonetic development) and/or many phonological errors and/or strongly modified sound of voice (e.g. nasality) that affects comprehensibility severely | Almost incomprehensible spontaneous speech |
| Syntactic and Morphological Skills (based on Clahsen, 1988) | Inconspicuous syntax (correct position of verbs in main- and subordinate clauses) and morphology (correct usage of morphological rules: gender-, case- and tense-marking; subject-verb congruency and plurals are used without errors) | Minor morphological abnormalities (e.g. impaired dative marking), syntactical rules are reliably mastered (correct position of verbs in main- and subordinate clauses) | Frequent morphological abnormalities (considerable difficulties in case marking, conspicuous subject-verb congruency); syntactical rules are generally mastered | Many morphological abnormalities and/or word order is not used systematically and/or reduced and conspicuous usage of function words (prepositions, conjugation, pronouns) | Many morphological abnormalities, unsystematic word order, strongly reduced usage of function words (prepositions, conjugation, pronouns) | Absent development of syntactic and morphological abnormalities, variable word order, no word inflection |
| Semantics and Lexicon (based on Glück, 2011) | Very good development of lexicon; good and flexible ability to formulate; Test for Lexicon and Word Finding (WWT): Above average performance in the highest age-norms (t-value >60) or very good development according to hearing age (t-value 50-60) | Minor abnormalities in development of lexicon; WWT: good results in relation to age-norm from 10 to 12 years (t-value 40-60) or inconspicuous results according to hearing age (t-value: 50-40) | Abnormalities in vocabulary development; formulations without variations; WWT: conspicuous results in relation to age norm 8-10 years or conspicuous results according to hearing age (t-value < 40) | Reduced vocabulary development for content- and function words, strong abnormalities for low-frequency words;  WWT: conspicuous results (t-value < 40 in the age norm 6-8 years) or significantly below average in hearing age adjusted norms | Only usage of high frequent function and content words | Rudimentary vocabulary; Almost exclusive use of nouns |
| Receptive Language (based on Fox, 2011) | Inconspicuous TROG-D: highly developed comprehension of case markings and complex sentences | Minor difficulties in language comprehension, only slightly noticeable during conversation. TROG-D: able to comprehend complex sentence structures, understanding of personal pronouns in accusative and dative and double-object-constructions are possible | Mild difficulties in language comprehension, TROG-D: able to comprehend passive sentences and simple sentences | Frequent difficulties in language comprehension TROG-D: able to comprehend longer main clauses and dependent clauses in first position (because-sentences) | Highly impaired in language comprehension; able to comprehend simple SVO sentences | Comprehension of single words; meaning is deducted from context; unable to comprehend conversations about topics not grounded in the current situation |

References:

Clahsen. 1988. Normale und gestörte Kindersprache. Linguistische Untersuchungen zum Erwerb von Syntax und Morphologie. (Benjamins J, editor.). Amsterdam

Fox. 2003. Kindliche Aussprachestörungen - Phonologischer Erwerb, Differenzdiagnostik, Therapie. 5th ed. Schulz-Kirchner

Fox. 2011. TROG-D - Test zur Überprüfung des Grammatikverständnisses. 5th ed. Schulz-Kirchner

Glück. 2011. Wortschatz- und Wortfindungstest für 6- bis 10-Jährige. 2nd ed. Urban & Fischer Verlag/Elsevier GmbH
