# Supplementary material for: Neural Correlates of Speech Processing in Prelingually Deafened Children and Adolescents with Cochlear Implants
Source: PLoS One. 2013 Jul 4;8(7):e67696. doi: 10.1371/journal.pone.0067696 (PMC3701579; doi:10.1371/journal.pone.0067696)
Supplement: Table S2 — Subjective Rating of Hearing. (DOCX) [file pone.0067696.s002.docx]

# Subjective rating of hearing

(According to the Manchester Teens Questionnaire: Post-operative)

|  | never | rarely ever | sometimes | often | always |
| --- | --- | --- | --- | --- | --- |
| Are you able to discriminate sounds with your CI only by listening? (E.g. a car, a phone, a door bell, a barking dog)? |  |  |  |  |  |
| Do you understand family and friends only by listening? |  |  |  |  |  |
| Do you understand unknown persons / strangers only by listening? |  |  |  |  |  |
| Do you easily understand people in noisy surroundings? |  |  |  |  |  |
| Do you understand what is said in a group? |  |  |  |  |  |
| Are you able to take part in a conversation in a group? |  |  |  |  |  |
| Do you feel at ease when talking to people that you do not know very well? |  |  |  |  |  |
| Do you contact your friends and family on the phone? |  |  |  |  |  |
| Do you contact unknown persons on the phone? |  |  |  |  |  |
| Do you easily understand telephone talk? |  |  |  |  |  |
| Do you like watching TV? |  |  |  |  |  |
| Do you like listening to music? |  |  |  |  |  |
